# Phylogeography of *Quercus variabilis* Based on Chloroplast DNA Sequence in East Asia: Multiple Glacial Refugia and Mainland-Migrated Island Populations

**DOI:** 10.1371/journal.pone.0047268

**Published:** 2012-10-24

**Authors:** Dongmei Chen, Xianxian Zhang, Hongzhang Kang, Xiao Sun, Shan Yin, Hongmei Du, Norikazu Yamanaka, Washington Gapare, Harry X. Wu, Chunjiang Liu

**Affiliations:** 1 School of Agriculture and Biology and Shanghai Jiao Tong University Research Centre for Low Carbon Agriculture, Shanghai Jiao Tong University, Shanghai, China; 2 Key Laboratory for Urban Agriculture (South), Ministry of Agriculture, People’s Republic of China, Shanghai, China; 3 Arid Land Research Center, Tottori University, Tottori, Japan; 4 Commonwealth Scientific and Industrial Research Organisation Plant Industry, Canberra, Australia; 5 Umeå Plant Science Centre, Swedish University of Agricultural Sciences, Umeå, Sweden; Roehampton University, United Kingdom

## Abstract

The biogeographical relationships between far-separated populations, in particular, those in the mainland and islands, remain unclear for widespread species in eastern Asia where the current distribution of plants was greatly influenced by the Quaternary climate. Deciduous Oriental oak (*Quercus variabilis*) is one of the most widely distributed species in eastern Asia. In this study, leaf material of 528 *Q. variabilis* trees from 50 populations across the whole distribution (Mainland China, Korea Peninsular as well as Japan, Zhoushan and Taiwan Islands) was collected, and three cpDNA intergenic spacer fragments were sequenced using universal primers. A total of 26 haplotypes were detected, and it showed a weak phylogeographical structure in eastern Asia populations at species level, however, in the central-eastern region of Mainland China, the populations had more haplotypes than those in other regions, with a significant phylogeographical structure (*N*_ST = _0.751> *G*_ST = _0.690, P<0.05). *Q. variabilis* displayed high interpopulation and low intrapopulation genetic diversity across the distribution range. Both unimodal mismatch distribution and significant negative Fu’s *F_S_* indicated a demographic expansion of *Q. variabilis* populations in East Asia. A fossil calibrated phylogenetic tree showed a rapid speciation during Pleistocene, with a population augment occurred in Middle Pleistocene. Both diversity patterns and ecological niche modelling indicated there could be multiple glacial refugia and possible bottleneck or founder effects occurred in the southern Japan. We dated major spatial expansion of *Q. variabilis* population in eastern Asia to the last glacial cycle(s), a period with sea-level fluctuations and land bridges in East China Sea as possible dispersal corridors. This study showed that geographical heterogeneity combined with climate and sea-level changes have shaped the genetic structure of this wide-ranging tree species in East Asia.

## Introduction

The climatic changes of the Quaternary (i.e., over the past 2.6 million years) have exerted a profound influence on the patterns of modern plant distribution and evolution at the global scale [1], [2]. Typical responses of plants to such climate changes were adaptive evolution through migration, resulting in the alteration of geographical distribution [3], [4]. For example, European deciduous oaks (*Quercus* spp.) retreated to several refugia in the Iberian Peninsula, Italy and the Balkans during the Last Glacial Maximum (LGM, between 26,500 and 19,000 years ago) period. During the inter-glacial periods, they recolonized the northern areas from these refugia [5], [6].

Similarly, East Asia experienced strong climate oscillations during the glacial and interglacial periods although no massive glaciers occurred since the Quaternary [7], [8]. A general view is that during the LGM period, the temperature declined by about 5–11°C relative to the current climate across the Asian Continent. After the LGM, the climate became warmer with several smaller glaciers occurring in an interval manner, resulting in decrease in temperature across the Asian continent [8]. Correspondingly, the range of vegetation experienced repeatedly retractions and expansions in eastern Asia [9]. There are at least two hypotheses proposed for the large scale change of vegetation in the East Asia. The steppe-desert hypothesis (SD-Hypothesis) proposes that at the LGM time, the steppe or desert vegetation reached the areas that was >31°N in latitude, resulting in the temperate deciduous forests being distributed from latitude 22°N to about 31°N, whereas the warm-temperate evergreen forests retreated southward (<22°N) in southeast China [8]–[10]. The second hypothesis of the multiple tree-refugia (MR-Hypothesis) postulates that multiple glacial refugia for tree species widely existed across northern and eastern Asia [11]–[13].

Relative to European and North American continents, eastern Asia showed a higher diversity of plant species due to unique topography and climate of the eastern Asia region since Quaternary [11]. The coastal East Asian areas experienced milder climate compared to inland (western) Asian areas, which gave rise to a distinct vegetation zone along the coastal areas of Mainland China covering the southwest to northeastern. Other areas that experienced similar mild climate included the Archipelagoes and Islands (e.g., Korean Peninsula, Zhoushan and Japanese Archipelagoes, and Taiwan Islands). Thus, eastern Asia is an ideal area for studying phylogeographical relationship, gene flow, and species distributions [14], [15].

With molecular data coupled with coalescent theory, distributional and demographic change through evolutionary time and the identification of glacial refugia have been conducted for *Ostryopsis davidiana* Decne in north China [13], *Ginkgo biloba* in south China [16], *Juniperus przewalskii* in northeast Qinghai-Tibetan Plateau [17], as well as for *Cyclobalanopsis glauca* in Taiwan Island [18], and *Aucuba japonica* [19] and *Aristolochia kaempferi* [20] in Japan. Qiu et al. [14], [15] examined the timing of genetic divergence for two *Kirengeshoma* species distributing in eastern China, southern Korea and Japan. However, the distribution ranges of these species do not cover the large part of eastern Asia, and it would be an advantage to examine how Quaternary climate impacted the retreat, migration, and re-colonization of a plant species distributed across the eastern Asia.

**Figure 1:**
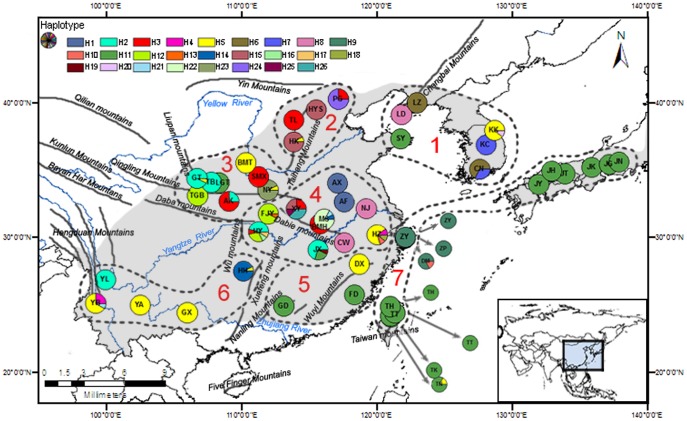
Distribution of 50 *Q. variabilis* populations (codes in Table 1) and 26 cpDNA haplotypes. The insert in the right bottom corner shows an overview of study area. The light gray shadow area is the current natural distribution of *Q. variabilis* [29], [73].

Oriental oak (*Quercus variabilis*) is one of the most widely distributed deciduous species in eastern Asia (latitude from 24° to 42° N and longitude from 96° to 140°E), and it covers Mainland China, Zhoushan Archipelago, Taiwan Island, Japanese Archipelago, and Korean Penninsula (Fig. 1). Little is known about the effect of past climate changes on the distribution of *Q. variabilis*. In this study we examined in detail the phylogeographical relationships between *Q. variabilis* populations and their possible evolutionary relationships. The species spans from temperate to subtropical zones and from continent China to islands (e.g., Taiwan, Zhoushan and Japan) and is an ideal species to study possible climate impact on the species distribution. There have been some small-scale investigations of population diversity by using nuclear gene or plasma gene marker for the species. For example, population genetic diversity of *Q. variabilis* has been investigated within Shaanxi and Henan provinces using isozyme [21]. Zhou et al. reported relatively higher genetic diversity within the three natural populations in northwest China of Shaanxi province, but lower gene diversity between the populations using the isozyme markers [22]. It was proposed that the current northern subtropical area was the central part of *Q. variabilis* distribution [21], [22]. However, population structure and genetic diversity have not been examined across the entire species distribution range, including peripheral populations in Archipelagoes of Zhouzhan and Japan, Taiwan Island, and Korean Penninsula by using more stable and informative DNA markers.

In 2008, we collected the samples of 50 *Q. variabilis* populations across species whole distribution and sequenced three cpDNA intergenic spacer fragments using leaf tissues. We specifically aimed to address the following questions: 1) how the identified haplotypes are distributed within and among populations of *Q. variabilis*; 2) how genetic variation is distributed between and within populations; 3) is there a phylogeographic relationship among the populations, in particular, between the populations growing in the Mainland China and the peripheral populations in the Archipelagoes and Island populations in Zhoushan, Japan and Tawain, and 4) what is possible paleodistribution and population demographic history of *Q. variabilis* in pan-eastern Asia. With information above, we could deduce the possible glacial refugia status in the eastern Asia during LGM period and explore the possible roles of land bridges in influencing geographic distribution of the species during the glacial period.

## Materials and Methods

### Sampling

A total of 50 natural *Q. variabilis* populations were selected across the entire natural distribution area of *Q. variabilis* in East Asia in 2008. Thirty-four populations were from Mainland China, three from Zhoushan Archipelago which located in East China Seas, four from Taiwan Island, three from Korea Peninsula and the remained six from Japanese Archipelago (Fig. 1; Table 1). At each population site, sampled trees were selected at least 100 m apart. The latitude, longitude and elevation of each collection location were measured using an Etrex handheld GPS unit (Garmin, Taiwan). The fresh leaves collected from the sampled trees were stored into a plastic bag with silica gel. The leaves were then transported to the laboratory at the School of Agriculture and Biology at Shanghai Jiao Tong University (SJTU) and stored in the −80°C refrigerator. A total of 528 individual trees were sampled and analyzed for this study ranging from 3 individual trees in Guangdong province (GD) to 17 trees in Liaoning province (LZ) with average of 10.6 trees per sampling site (Table 1).

**Table 1:** Geographic origins, sample sizes (n), haplotype diversity (*H*d), nucleotide diversity (π×10^3^) and haplotypes of the 50 *Q. variabilis* populations sampled in eastern Asia^1^.

Code	Locations	Latitude (N)	Longitude (E)	Altitude (m)	Trees sampled	Haplotype diversity (*Hd*)	Nucleotide diversity (π×10^3^)	Haplotypes (no. of individuals)
**1. Northeastern China and Korean Peninsula**								
SY	Kunyu Mt, Yantai, Shandong	37°18′	121°45′	223	10	0.000	0.00	H11 (10)
LD	Dahei Mt, Dalian, Liaoning	39°06′	121°48′	180	10	0.000	0.00	H8 (10)
LZ	Zhuanghe, Dalian, Liaoning	39°59′	122°58′	250	17	0.000	0.00	H6 (17)
CN	Baekwoon Mt, Cheonnam, Korea	35°04′	127°36′	482	9	0.500	0.63	H6 (6), H7 (3)
KC	Wolak Mt, Chungbuk, Korea	36°51′	128°04′	335	12	0.000	0.00	H7 (12)
KK	Yangyang, Kangwon, Korea	37°56′	128°42′	487	9	0.222	0.28	H5 (8), H20 (1)
**2. Northern China**								
HX	Baian, Xingtai, Hebei	37°05′	113°50′	801	12	0.167	0.00	H5 (1), H15 (11)
TL	Tuoliang Scenic Area, Shijiazhuang, Hebei	38°41′	113°49′	1145	10	0.000	0.00	H3 (10)
HYS	Hongya Mt, Baoding, Hebei	39°29′	115°29′	516	12	0.000	0.00	H15 (12)
PG	Sizuolou Forest, Pinggu, Beijing	40°15′	117°07′	260	16	0.400	1.02	H3 (4), H24 (12)
**3. Northwestern China**								
AK	Xiangxidong, Ankang, Shaanxi	32°40′	109°02′	370	12	0.409	0.52	H2 (3), H3 (9)
TGB	Tuguanpu, Hanzhong, Shaanxi	33°06′	106°42′	715	4	0.000	0.00	H12 (4)
NY	Baotianman, Nanyang, Henan	33°30′	111°55′	1112	13	0.154	0.10	H5 (1), H23 (12)
LGT	Louguantai National Forest Park, Xi’an, Shaanxi	34°03′	108°16′	701	8	0.000	0.00	H11 (8)
TB	Taibai Mt, Baoji, Shaanxi	34°05′	107°42′	2007	10	0.000	0.00	H2 (10)
SMX	Ganshan Mt, Sanmenxia, Henan	34°30′	111°13′	1121	6	0.000	0.00	H3 (6)
BMT	Baimatan, Yan’an, Shaanxi	35°32′	110°16′	960	7	0.000	0.00	H5 (7)
GT	Dongcha Forest, Tianshui, Gansu	35°32′	110°07′	1028	13	0.154	0.10	H2 (12), H5 (1)
**4. Central-Eastern China**								
JX	Yunshan Reclamation Field, Yongxiu, Jiangxi	29°05′	115°37′	360	13	0.500	0.88	H2 (9), H11 (3), H19 (1)
CW	Chawan Forest, Huangshan, Anhui	29°36′	117°33′	459	8	0.000	0.00	H8 (8)
HZ	West Tianmu Mt, Hangzhou, Zhejiang	30°12′	120°00′	349	10	0.533	0.23	H4 (1), H5 (7), H10 (1), H18 (1)
HY	Yichang, Hubei	30°26′	111°12′	276	11	0.764	0.69	H2 (5), H3 (1), H12 (3), H16 (1), H17 (1)
BMH	Baimiaohe, Luotian, Hubei	31°01′	115°46′	312	14	0.143	0.18	H3 (13), H4 (1)
MS	Maoshan Forest, Huoshan, Anhui	31°21′	116°05′	659	12	0.318	0.38	H14 (1), H21 (1), H22 (10)
FJY	Fangjiaya, Nanzhang, Hubei	31°45′	111°56′	237	13	0.154	0.00	H12 (12), H13 (1)
XY	Nanwan Scenic Area, Xinyang, Henan	32°07′	114°00′	131	8	0.821	1.11	H3 (2), H15 (2), H25 (1), H26 (3)
NJ	Xiashu Forest, Jurong, Jiangsu	32°08′	119°12′	160	10	0.000	0.00	H8 (10)
AF	Fengyang, Anhui	32°39′	117°34′	28	12	0.000	0.00	H1 (12)
AX	Huangzangyu National Park, Xiaoxian, Anhui	34°01′	117°03′	117	11	0.000	0.00	H1 (11)
**5. Southeastern China**								
DX	Dangxi, Nanping, Fujian	28°02′	118°41′	704	7	0.000	0.00	H5 (7)
FD	Dehua, Quanzhou, Fujian	25°45′	118°19′	484	12	0.000	0.00	H11 (12)
GD	Nanling National Forest Park, Guangdong	24°55′	113°05′	500	3	0.000	0.00	H11 (3)
**6. Southwestern China**								
GX	Pinglou, Tianlin, Guangxi	24°26′	105°56′	696	11	0.000	0.00	H5 (11)
YA	Wenquan Town, Anning, Yunnan	24°59′	102°27′	1826	15	0.000	0.00	H5 (15)
YB	Baoshan Mt, Baoshan, Yunnan	25°07′	99.°28′	1821	12	0.530	0.31	H4 (3), H5 (8), H21 (1)
YL	Shigu Town, Lijiang, Yunnan	26°52′	99.°40′	112	8	0.000	0.00	H2 (8)
HH	Kang Long Nature Reserve, Huaihua, Hunan	27°31′	110°06′	455	14	0.143	0.09	H5 (1), H14 (13)
**7. Zhoushan and Japanese Archipelagoes and Taiwan Island**								
DM	Damao Island, Zhoushan, Zhejiang	29°58′	122°03′	92	11	0.327	0.42	H9 (9), H10 (2)
ZP	Panzhi, Zhoushan, Zhejiang	29°59′	122°04′	84	12	0.000	0.00	H9 (12)
ZY	Yancang, Zhoushan, Zhejiang	30°02′	122°05′	42	12	0.000	0.00	H9 (12)
TN	Shou-Cheng Mt, Nantou, Taiwan	24°05′	121°02′	756	6	0.333	0.42	H5 (1), H11 (5)
TK	Guguan, Taichung, Taiwan	24°12′	120°60′	750	9	0.000	0.00	H11 (9)
TT	Wuling Farm, Taoshan, Taiwan	24°24′	121°18′	1910	11	0.000	0.00	H11 (11)
TH	Kengzihkou Range, Hsinchu, Taiwan	24°53′	120°58′	100	14	0.000	0.00	H11 (14)
JY	Gabizan Mt, Yamaguchi, Japan	33°56′	131°58′	99	7	0.000	0.00	H11 (7)
JT	Okayama, Japan	34°43′	133°54′	200	11	0.000	0.00	H11 (11)
JH	Kamagamine Mt, Hiroshima, Japan	34°56′	132°56′	511	12	0.000	0.00	H11 (12)
JK	Experimental Plot of Lake Biwa, Kyoto, Japan	35°11′	135°54′	193	12	0.000	0.00	H11 (12)
JG	Matsuno Lake, Gifu, Japan	35°25′	137°11′	208	8	0.000	0.00	H11 (8)
JN	Iida, Nagano, Japan	35°35′	137°56′	473	9	0.000	0.00	H11 (9)

1 50 sites were grouped into seven regions and their locations were depicted in the Fig. 1.

### DNA Extraction, Polymerase Chain Reaction (PCR) and Sequencing

Approximately 50 mg silica gel-dried leaves were grounded using Precellys 24 tissue homogenizer (Bertin Technologies, France). Genomic DNA was extracted from leaf powder using modified hexadecetyltrimethyl ammonium bromide (CTAB) procedure based on Doyle’s [23]. DNAs were dissolved in a 50 µl TE buffer for storage, and the concentration and quality of the extracted DNA was checked by running aliquots on a 1% agarose gel electrophoresis with a TaKaRa DL2000 marker. Twelve universal primers were initially randomly amplified and sequenced in intergenic regions to reveal sequence variation among *Q. variabilis*. Three intergenic spacer (IGS) primes (*trn*L-*trn*F, *atp*B-*rbc*L, *trn*H-*psb*A) were selected according to sequencing performance and variation results (Information S1). Polymerase chain reaction (PCR) was used to isolate and amplify the noncoding region of cpDNA. PCR reactions contained 1 µl of the DNA extraction, 5 µl of 10 × PCR buffer containing 1.5 mM MgCl_2_, 10 mM Tris-HCl (PH 8.3), 50 mM KCl, 0.2 µM of each primer, 200 µM of each dNTP, 1.25 U of Taq polymerase (TaKaRa) in a total volume of 50 µl. PCR was conducted on a Mastercycler pro Thermal Cycler (Eppendorf, Germany), and performed with initial denaturing of 5 min at 94°C followed by 35 cycles of 30 s at 94°C, 30s of annealing at 55°C, 90 s of elongation at 72°C, ending with a 10-min extension at 72°C. The products were checked on a 1% agarose gel, and then the products with a single band were directly sequenced in both directions by standard methods using a Taq dye deoxy terminator cycle sequencing kit on an Applied Biosystems Model 3730xl automated sequencer (Applied Biosystems, USA).

### Phylogeographical and Population Genetic Analyses

Multiple alignments of the sequences were obtained using the program CLUSTALW [24] with subsequent manual adjustment. All sequences were deposited into GenBank with accession numbers JF753573-JF753598. To measure the level of genetic variation, average pairwise differences per base-pair between sequences (nucleotide diversity, π) [25] and haplotype diversity (*H*d) were calculated using DnaSP v5 [26]. An un-rooted network was constructed by coalescent simulations using the Median-Joining model implemented in NETWORK version 4.6 [27], to view intraspecific relationships between the cpDNA haplotypes.

In order to estimate genetic differentiation among the populations from different geographic regions, the distribution range of *Q. variabilis* populations was divided into seven regions (marked from 1 to 7 in Table 1 and Fig. 1) mainly based on geographical and climatic vicinity [28]. Considering their similar flora [29], [30], Liaotung Peninsular and Korea Peninsular were grouped into the same region (region 1) and the islands, such as Japan, Zhoushan and Taiwan, into the same region (region 7, Fig. 1). Average gene diversity within a population (*H*_S_), total gene diversity (*H*_T_) and two parameters of population differentiation, *G*_ST_ (coefficient of genetic variation over all populations) [31] and *N*_ST_ (coefficient of genetic variation influenced by both haplotype frequencies and genetic distances between haplotypes), were estimated by the program PERMUT (available at www.pierroton.inra.fr/genetics/labo/Software/) [32]. A permutation test with 1000 permutations was used to compare the difference between *N*_ST_ than *G*_ST_. A higher *N*_ST_ than *G*_ST_ usually indicates the presence of phylogeographic structure with similar (closely related) haplotypes, being found more often in the same area than less similar haplotypes [17]. The amount of variation among populations within a region and within a population was calculated by the hierarchical analysis of molecular variance (AMOVA) framework carried out using ARLEQUIN (version 3.5) [33], and significant difference was tested by a nonparametric permutation procedure with 1000 permutations. Population differentiation was also quantified with nonhierarchical analysis of molecular variance by estimating *F*_ST_ between mainland and islands populations and among islands populations.

Isolation by distance (IBD) [34] under a two-dimensional stepping-stone model [35] was tested using the matrix correlation method of Mantel implemented in IBD 1.52 [36]. *F*_ST_/(1−*F*_ST_) was calculated in ARLEQUIN and geographical distances were estimated with the program FOSSIL, and implemented in R language.

### Phylogenetic Divergence Time Estimation and Demographic Analyses

Phylogenetic evolutionary tree of haplotypes was created by BEAST 1.7.3 which is a Bayesian statistical framework [37]. Based on nucleotide substitutions and cpDNA alignment, parameter of BEAST was HKY substitution model selected by jModelTest 0.1.1 [38], an uncorrelated lognormal relaxed clock with the fossil data of deciduous *Quercus* [39] and a Bayesian Skyline coalescent tree prior. The tmrca of tree prior was reseted by fossil data mean ± stdev (5.5±1.2 Ma) as normal distribution. In this analysis, MCMC were run for 1×10^7^ generations, with sampling every 2000th generation, following a burn-in of the initial 10% cycles. The phylogenetic evolutionary tree was edited in FigTree v1.3.1 software (http://tree.bio.ed.ac.uk/software/figtree).

Tajima’s *D* [40] and Fu’s *F_S_* [41] tests were performed to discriminate mutation/drift equilibrium and to evaluate the hypothesis of population expansion through the significant excess of low-frequency haplotypes. For neutral markers, significant negative values of *D* and *F* can be expected in cases of population expansion. Based on phylogeographical structure results, the demographic history of both populations in Central-Eastern China and East Asia was investigated using mismatch distribution analysis (MDA) in ARLEQUIN, to assess whether intraspecific lineages experienced past population expansions. The sum of square deviations between the observed and expected distributions and the raggedness index of the observed distribution were used as test statistics to validate fit of the models [42], [43].

In addition, we used two methods to estimate the time of *Q. variabilis* population demographic expansion. First, the expansion time was estimated directly from the mismatch distribution with the statistic τ (tau) and translated into absolute time in years (t), using the equation *T* = τ/2u [42], [44], where *u* is the neutral mutation rate for the entire sequence per generation and is calculated as *u* = *µ*kg, where *µ* is the substitution rate in substitutions per site yr^−1^ (s/s/y), k is the average sequence length of the cpDNA region under study (here, 1670 bp), and g is the generation time in yr (here, 50 years). The mean substitution rate of the three combined cpDNA-IGS regions was obtained from the corresponding clock-calibrated BEAST tree of *Q. variabilis*. Second, we employed coalescence-based skyline-plot method to date population size changes over time (Bayesian skyline plot: BSP) using the BEAST program and Tracer v1.5 software (http://tree.bio.ed.ac.uk/software/tracer/).

### Ecological Niche Modelling to Construct Palaeodistribution

Maximum entropy modelling technique was employed to construct pontential palaeodistribution of *Q. variabilis* via MAXENT (version 3.3.3 k) [45]. This approach is considered advantageous because it is not biased by limited absence records [46], although it does assume that preferences for climatic conditions do not change over time. Information on the geographic distribution of *Q. variabilis* was based on 325 presence records in the Chinese mainland sourced from Chinese Virtual Herbarium (http://www.cvh.org.cn/cms/) and 150 occurrence records in Taiwan, Japan, and Korea obtained from Global Biodiversity Information Facility (http://data.gbif.org/welcome.htm), after the removal of duplicate and inexistent records according to field surveys within each pixel (2.5 arc-min; 5 km). Bioclimatic variables were downloaded from the WorldClim database [47] for current conditions and for the LGM at 2.5′ spatial resolution. LGM data were obtained from two different general circulation model (GCM) simulations: the Community Climate System Model (CCSM) [48] and the Model for Interdisciplinary Research on Climate (MIROC) [49], which are available as downscaled high-resolution estimates of LGM climate parameters [47]. Six variables were chosen with a consideration of ecological significance and to avoid having to include highly correlated variables (data not shown), and thus prevent potential overfitting [50]: annual mean temperature, isothermality, max temperature of warmest month, annual precipitation, precipitation of wettest month, and precipitation of driest month. Model validation was performed using default settings with 10 replicates and 25% of the data used for model testing, while a regularization multiplier of 3 was chosen. Model accuracy was evaluated by assessing the area under the curve (AUC) of the receiver-operating characteristic (ROC) plot [45], a threshold-independent index widely used in ecological studies that ranges from 0.5 (randomness) to 1 (exact match), where scores between 0.7 and 0.9 indicate good discrimination [51]. Cut-off thresholds for areas predicted as ‘suitable’ were determined according to logistic threshold maximum training sensitivity plus specificity as provided by MAXENT. Although differences between the two model outputs for the LGM (CCSM and MIROC) are interesting, we combined with molecular data and the estimated ecological niche on to the reconstructed LGM climatic conditions simulated by MIROC model was selected. The present and past distribution modelling results were presented while seafloor topography data (ETOPO1) from the National Geophysical Data Center of National Oceanic and Atmospheric Administration (NOAA,USA) were used to estimate the palaeocoast lines (-130 m than at present) and the palaeoclimate surfaces of the exposed seafloor area during the LGM [52].

## Results

### Haplotype Distribution and Phylogenetic Analyses

The three cpDNA IGS regions surveyed across the 528 individuals of *Q. variabilis* were aligned along a total length of 1670 bp with 23 variable nucleotide sites observed (Information S1). A total of 26 haplotypes were detected in the 50 *Q. variabilis* populations across eastern Asia (Information S1, Fig. 1). Of the 26 haplotypes, 15 were shared by at least two populations while the other 11 haplotypes were only found in a single population (Information S1). The most common haplotypes were H11 (found in 15 populations with a frequency of 0.254) and H5 (found in 12 populations with a frequency of 0.129). H11 was found in all Japanese Archipelago and Taiwan Island populations and some coastal populations of Mainland China (e.g., GD and FD in Southeastern China and SY in Shandong provinces), and only in two inland populations of Mainland China (JX in Central-Eastern China, and LGT in Northwestern China) (Information S1). H5 was more widely distributed across China and Korea (Information S1), but by contrast mainly observed in the inland populations of the Mainland China (e.g., HX in Northern China, NY, BMT, and GT in Northwestern China, HZ in Central-Eastern China, DX in Southeastern China) with a concentration in Southwestern China (e.g., GX, YA, YB and HH). In contrast to H11, H5 was rarely found in the Archipelagoes and Islands populations (only in population TN of Taiwan).

HY population in the west of Hubei province in China had the highest number (five) of haplotypes (H2, H3, H12, H16 and H17) while population XY located in Dabie Mountains, Henan province and HZ in Zhejiang province had four haplotypes (H3, H15, H25 and H26) (Table 2). The populations having three haplotypes were JX in Jiangxi province (H2, H11, and H19), MS in Anhui province (H14, H21, and H22) and YB in Yunnan province (H4, H5, and H21). All these populations were located in Central-Eastern China Region except for population YB which is from Southwestern China. A noteworthy point is that widely-distributed H5 and H11 haplotypes were not found in the population HY. However, haplotypes found in HY population were similar to those found in Northwestern, North and Central China’s populations (H2, H3 and H12, Information S1).

**Table 2:** Divergence (*F*_CT_) between *Q. variabilis* populations in the Mainland China, Archipelagoes, Taiwan Island and Korean Peninsula in eastern Asia.

*F*_CT_	Zhoushan Archipelago	Taiwan Island	Korea Peninsula	Japan Archipelago
Mainland China	0.821	0.828	0.817	0.832
Zhoushan Archipelago	1	0.094	0.343	0.076
Taiwan Island		1	0.553	0.084
Korea Peninsula			1	0.584
Japan Archipelago				1

Note: *F*_CT_ means the fixation index of genetic variation between the populations in the mainland and islands.

**Figure 2:**
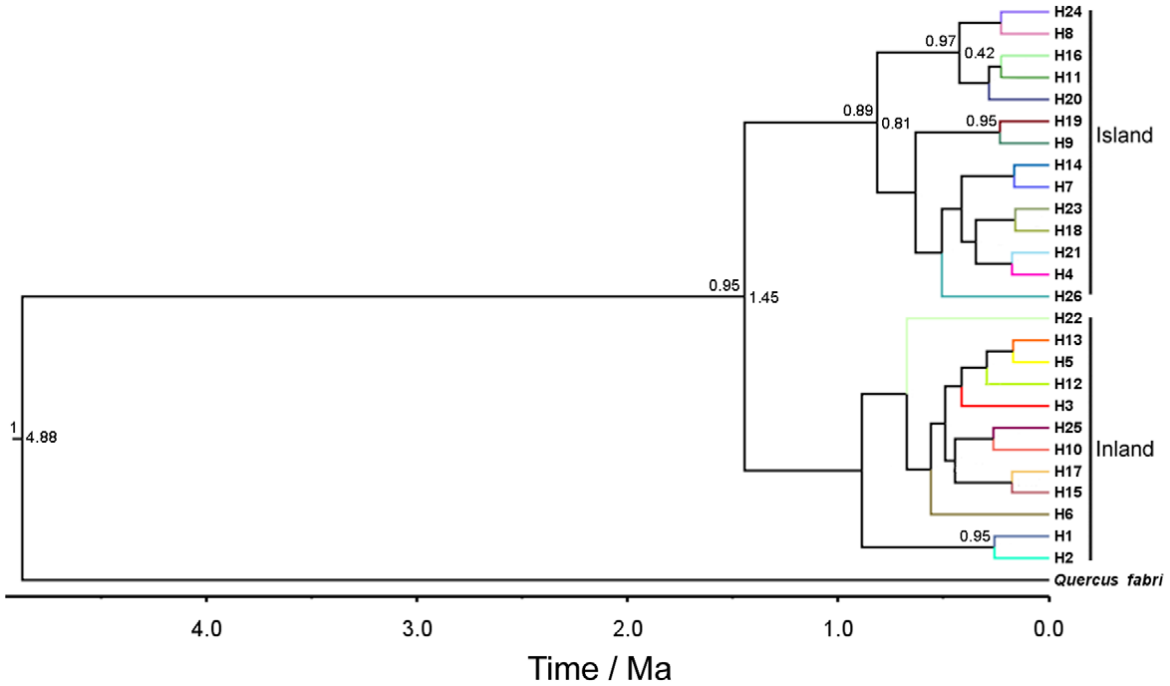
BEAST-derived chronograms of 26 haplotypes of *Q. variabilis*. The numbers above the branches are posterior probabilities (PP>0.6). Node ages are labeled in the nodes selectively based on posterior probabilities.

H6 existed only in Liaotung Peninsular and Korea Peninsular whereas H10 was only found in Zhejiang province, and H1 occurred only in populations from Anhui province (AX, AF). The Bayesian phylogenetic tree was shown in Fig. 2, which was divided into two major clades. The phylogenetic tree does not resolve all the evolutionary relationships of haplotypes and showed polytomies structure that might have stemmed from rapid population radiations. The rare haplotypes H7, H9, and H20 and common haplotype H11 were observed in the populations from Korea Peninsula, Zhoushan Archipelago, and Taiwan and Japan Islands, with other haplotypes mainly found in coastal and central areas were clustered into one clade (marked as ‘Island’ clade). The endemic haplotypes H6 and H10 which were shared between mainland and island populations were clustered with other mainland only haplotypes into the second clade (marked as ‘Inland’ clade).

**Figure 3:**
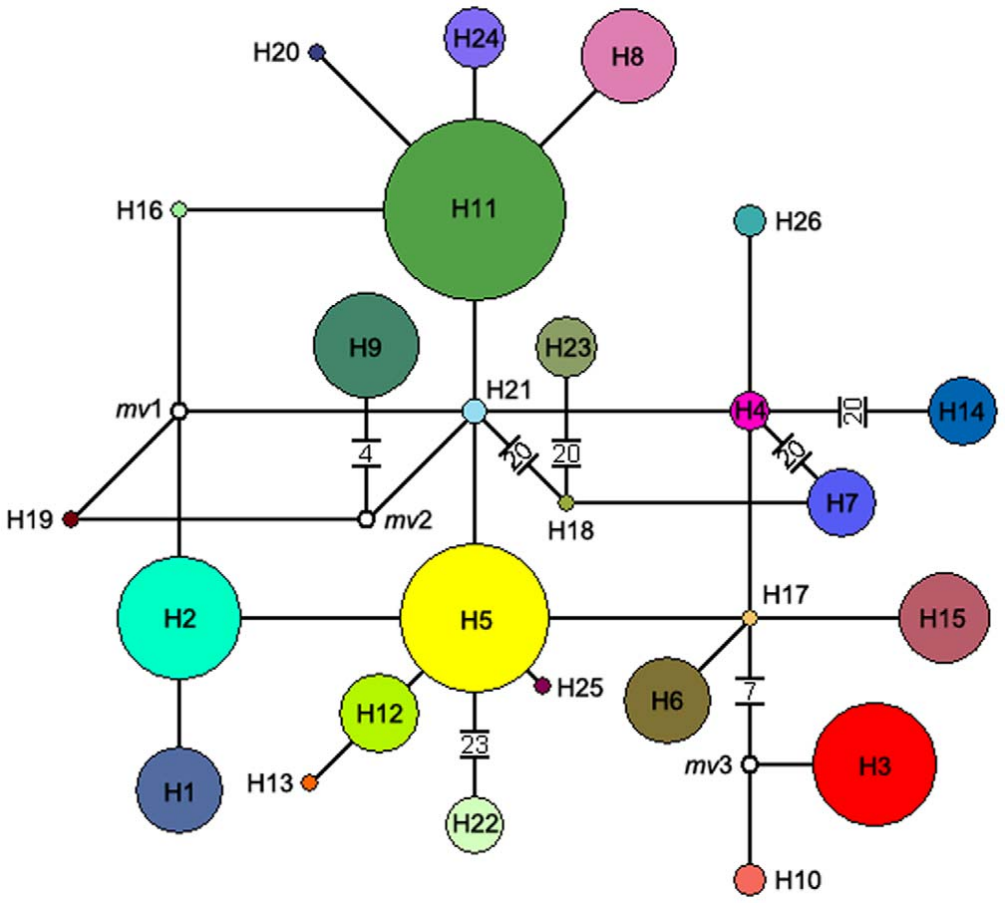
The phylogenetic network of 26 cpDNA haplotypes of *Q. variabilis*. Circle size is proportional to the frequency of a haplotype over all the populations, with the largest circle representing the most abundant haplotype. Each line between haplotypes represents a mutational step; the number noted between two parallel bars indicates the number of hypothetical missing haplotypes. The small solid white circles represent existing un-sampled haplotypes or extinct ancestral haplotypes.

A phylogenetic network was established among 26 haplotypes (Fig. 3). The network did not show any significant geographical clades. Three median vectors (*mv*) in the network frame can be recognized in the network that *mv*1 connects H2, H16, H19 and H21, *mv*2 connects H9, H19 and H21, and mv3 connects H3, H10 and H17. These median vectors might be an indication that the three genotypes were missing in the sample collection during the sampling process, or were ancestor genotypes which had been extinct [53]. The exceptionally large number of mutational steps (>20) separating Korean haplotype H7 from H4 in China mainland points to a long period of time for which the haplotypes evolved separately. H9 exists in Zhoushan archipelago probably evolved from an ancestral haplotype existed in mainland. A ‘star-like’ phylogeny of haplotypes would be expected for a scenario of rapid range expansion.

### Genetic Diversity and Phylogeographical Structure

Within Mainland China, the central parts contain the *Q. variabilis* populations with more haplotypes and higher nucleotide diversity (Table 1). Populations XY (Henan province) and HY (Hubei province) had the highest haplotype diversity (*H*d = 0.821 and 0.764, respectively) and nucleotide diversity (*π* = 1.11 and 0.69×10^−3^, respectively, Table 1). Both XY and HY population were from the central part of the Central-Eastern China region in the area between Yellow and Yangtze rivers (Fig. 1). Other populations from the same Central-Eastern China region also showed high nucleotide diversity (JX, *π* = 0.88×10^−3^) or haplotype diversity (HZ, *H*d = 0.533). Population PG, from Beijing in Northern China (about 1000 km north of the Central China region, with the value of *H*d = 0.4 and *π* = 1.02×10^−3^) and YB, from Yunnan province in Southwestern China (with *H*d = 0.53 and *π* = 0.31×10^−3^, Table 1) are a few populations exceptions with high genetic diversity, but not located in the highest diversity Central-Eastern China region. For the populations in the Archipelagoes, Island and Korean Peninsula, population CN (Cheonnam, Korea with *H*d = 0.50 and *π* = 0.63×10^−3^) had the highest haplotype diversity and nucleotide diversity, population TN (Nantou, Taiwan with *H*d = 0.333 and *π* = 0.42×10^−3^) and DM (Zhoushan, China with *H*d = 0.327 and *π* = 0.42×10^−3^) had the second highest diversities, while all other populations (including all Japanese Archipelago populations) had low or zero diversity (Table 1).

The total genetic diversity for all populations (*H*_T_) was high (0.888±0.0284, Table 3). Average intra-population genetic diversity was estimated as *H*_S = _0.131±0.03, and average inter-population genetic diversity (G_ST = _0.852±0.03, Table 3). Intra-population genetic diversity (*H*_S_) was essentially zero in populations from Southeastern China and 0.294±0.09 for populations from Central-Eastern China. By contrast, inter-population differentiation was the lowest in populations from Central-Eastern China (*G*_ST_ = 0.690±0.095) relative to populations from other regions (for example, *G*_ST_ = 0.902±0.06 in Northwestern China).

**Table 3:** Estimates of *H*_S_, *H*_T_, *G*_ST_ *N*_ST_ and *N*m (mean ± se in parentheses) within regions.

Regions	Population No.	*H*_T_ (se)	*H*_S_ (se)	*G*_ST_ (se)	*N*_ST_ (se)	*P*	*N*m
Northeastern China and Korean Peninsula		0.933 (0.0470)	0.120 (0.0842)	0.871 (0.0972)	0.877 (0.0929)	0.442^ns^	0.074
Shandong Peninsula	SY	NC	NC	NC	NC	NC	
Liaoning province	LD/LZ	1.000 (0.0000)	0.000 (0.0000)	NC	NC	NC	
Korean Peninsula	CN/KC/KK	0.889 (0.1096)	0.241 (0.1446)	0.729 (0.1703)	0.719 (0.2040)	NS	0.186
Northern China	HX/TL/HYS/PG	0.806 (0.1196)	0.142 (0.0946)	0.824 (0.1048)	0.799 (0.1088)	NS	0.126
Northwestern China	AK/TGB/NY/LGT/TB/SMX/BMT/GT	0.917 (0.0464)	0.090 (0.0518)	0.902 (0.0602)	0.904 (0.0698)	0.441^ns^	0.054
Central-Eastern China	JX/CW/HZ/HY/BMH/MS/FJY/XY/NJ/AF/AX	0.947 (0.0339)	0.294 (0.0947)	0.690 (0.0954)	0.751 (0.0954)	0.012*	0.225
Southeastern China	DX/FD/GD	0.667 (0.2222)	0.000 (0.0000)	NC	NC	NC	
Southwestern China	GX/YA/YB/YL/HH	0.748 (0.1529)	0.135 (0.1027)	0.820 (0.1482)	0.839 (0.1079)	0.043^ns^	0.109
Zhoushan and Japanese Archipelagoesand Taiwan Island	DM/ZP/ZY/TN/TK/TT/TH/JY/JT/JH/JK/JG/JN	0.409 (0.1327)	0.051 (0.0344)	0.876 (0.0765)	0.840 (0.0961)	0.520^ns^	0.071
All data (excluding Archipelagoes and Islands)		0.927 (0.0147)	0.160 (0.0387)	0.828 (0.0416)	0.836 (0.0431)	0.208^ns^	0.104
All data		0.888 (0.0284)	0.131 (0.0306)	0.852 (0.0333)	0.855 (0.0350)	0.422^ns^	0.087

Note: Gene diversity within *Q. variabilis* populations (*H*_S_), total gene diversity (*H*_T_), interpopulation differentiation (*G*_ST_), and the number of substitution types (*N*_ST_) (mean ± se in parentheses) within regions and all combined calculated with PERMUT, using a permutation test with 1000 permutations. *N*m calculated by using *G*_ST_.

* indicates *N*_ST_ is significantly different from *G*_ST_; NS, not significantly different; NC, not computed due to a small sample size.

There was a significant difference in genetic diversity (*P* = 0.012) between *G*_ST_ (0.690) and *N*_ST_ (0.751) in Central-Eastern. This implied that there would be a significant phylogeographical structure in the Central-Eastern China region.

Hierarchical analysis of molecular variance (AMOVA) indicated that 79.5% variation was partitioned among populations and only 17.51% of the variation within populations (all partitions were significant at *P*<0.001) (Table 4). However, only 2.96% of the variation was partitioned among the seven different geographic regions.

**Table 4:** Hierarchical analysis of molecular variance (AMOVA) of *Q. variabilis* populations based on nucleotide sequences in eastern Asia.

Source of variation	d.f.	Sum of squares	Variance components	Percentage of variation	Fixation indices
Among the regional groups	6	52.076	0.028	2.96	*F*_CT_ = 0.030, *P*>0.1
Among the populations within the regional groups	31	249.700	0.750	79.52	*F*_SC_ = 0.820, *P*<0.001
Within the populations	357	58.995	0.165	17.51	*F*_ST_ = 0.825, *P*<0.001
Total	527	497.782	0.961		

Results from divergence test are presented in Table 2. Generally there was higher genetic differentiation between populations in Mainland China and populations in Zhoushan and Japanese Archipelagoes, Taiwan Islands, and Korean Peninsula (*F*_CT_ = 0.817–0.832). In contrast, genetic differentiation among populations within Zhoushan and Japanese Archipelagoes and Taiwan Islands was low (0.076–0.094, Table 2). A Mantel test conducted on the genetic and physical distance matrices found no significant correlation (r = 0.020; *P* = 0.318). This may indicate that isolation by distance effect was minimal in *Q. variabilis* populations in eastern Asia.

### Phylogeny-based Estimations of Divergence Times

The BEAST-derived cpDNA (*trn*L-*trn*F, *atp*B-*rbc*L, *trn*H-*psb*A) chronogram of *Q. variabilis* (Fig. 2) recovered a divergence event started from Pliocene (5.3 Ma-2.6 Ma BP). Based on this chronogram, we estimated the crown group age of *Q. variabilis* (PP = 1) as 4.88 Ma (95% highest posterior density, HPD: 2.24–7.41 Ma), and obtained a point estimate for the coalescent time (1.45 Ma, HPD: 0.31–3.18 Ma, PP = 0.95) of ‘Island’ clade and ‘Inland’ clade, suggesting a Quaternary split in haplotypes between Islands and Mainland in eastern Asia. H11, widespread in Taiwan and Japan Islands, was given a divergence time after 0.42 Ma (HPD: 0.04–1.29 Ma, PP = 0.97).

Based on the cpDNA-IGS chronogram of *Q. variabilis*, the BEAST analysis provided an average substitution rate of 7.905×10^−10 ^s/s/y. This is lower than the average values generally reported for non-coding regions of the chloroplast genome (e.g., 1.2–1.7×10^−9^ s/s/y) and the slowest reported cpDNA substitution rates for angiosperms (1.1×10^−9^ s/s/y), but consistent with the notion that woody taxa and/or phylogenetic relicts should have slower rates of molecular evolution [52], [54].

### Population Demographic History

Mismatch distribution analysis were conducted for all populations in East Asia and populations in Central-Eastern China where a phylogeographical structure was detected, respectively, and both were unimodal (figures not shown). East Asia populations show spatial and demographic expansions supported by uniformly significant SSD and *H*_Rag_ values. For Central-Eastern China populations, demographic expansions supported by significant SSD, spatial expansions supported by nonsignificant SSD and *H*_Rag_ values (Table 5). Tajima’s D were both nonsignificant positive, whereas, the Fu’s *F_S_* were both significant negative in whole and regional populations. As Fu [41] has noticed that the *F_S_* statistic was very sensitive to population demographic expansion, which generally leads to large negative *F_S_* values. The large negative Fu’s *F_S_* value support hypothesis that *Q. variabilis* in East Asia has experienced a demographic expansion. We dated the spatial expansion of *Q. variabilis* in East Asia to the period of the last glacial cycle(s) (16.7 kyr, 95% CI: 8.3–21.9 kyr, Table 5) based on the corresponding τ values and assuming a substitution rate of 7.905 ×10^−10 ^s/s/y estimated before.

**Table 5:** Summary of mismatch distribution parameters and neutrality tests for regional and East Asia *Quercus variabilis* populations.

Region	Model	Parameter (τ)	Expansion time (t) in kyr BP	SSD	*H*_Rag_	Fu’s *F_S_*	Tajima’s *D*	Mismatch Distribution
Central-EasternChina	Demographic expansion	2.242 (1.104–2.742)	17.0 (8.4–20.8)	0.007*	0.051^NS^	−9.829*	0.313^NS^	Unimodal
	Spatial expansion	2.253 (0.849–2.974)	17.1 (6.4–22.5)	0.006^NS^	0.051^NS^			
East Asia	Demographic expansion	2.164 (1.914–2.676)	16.4 (14.5–20.3)	0.018*	0.072*	−11.188*	0.156^NS^	Unimodal
	Spatial expansion	2.201 (1.095–2.886)	16.7 (8.3–21.9)	0.017*	0.072^NS^			

Estimates were obtained under models of spatial or pure demographic expansion using ARLEQUIN.

Note: *There is a significant difference at α = 0.05 level (Fu, 1997), and NS means no significant at α = 0.05 level.

**Figure 4:**
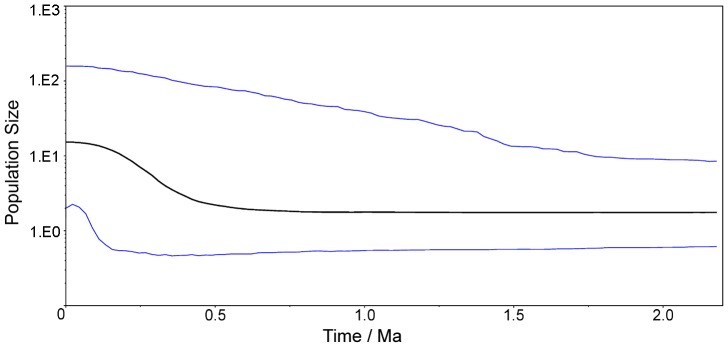
Bayesian skyline plot. The x axis is time of mutations per site before present, and the y axis is the expressed population size estimated in units of N_e_µ (N_e_: effective population size, µ: mutation rate per haplotype per generation), Dark line represents median inferred Neµ, blue lines mark the 95% highest probability density (HPD) intervals.

The Bayesian skyline plot (BSP) simulated the fluctuation of populations over time. Recent population size increases were observed in Middle Pleistocene (0.78 Ma - 0.13 Ma BP) from 0.5 Ma before present (Fig. 4). The result was robust, as different coalescent models in the BEAST analysis resulted in similar estimates.

### Palaeodistribution Modelling

**Figure 5:**
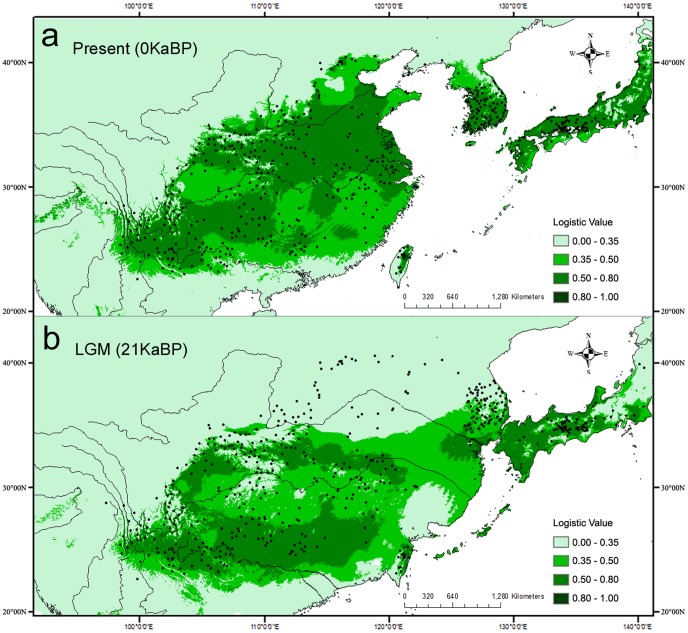
Ecological niche modelling. Predicted distribution probability (in logistic value) is shown in each 2.5 arc-min pixel, based on the palaeodistribution modelling at present (0BP) (a) and at the last glacial maximum (LGM) (21KaBP) (b). The distribution of river systems on the exposed East China Sea during the LGM was drawn from Shota et al. (2012). Occurrence records of *Q. variabilis* at present are also plotted as black points in the maps.

The test area under curve (AUC) for the ecological niche modelling (ENM), averaged across all 10 runs, was moderately high (0.879, standard deviation = 0.016, range = 0.863–0.895). The predicted distribution based on the model was similar to the species actual distribution (Fig. 5 a), with wide suitable ranges in East Asia. By contrast, the species distribution in most of China, southern Japan and the southern Korean Peninsula indicating a general southward range shift under the LGM climate (Fig. 5 b). Northern Mainland China, Korean Peninsula and Japan Islands were predicted as unsuitable habitats. The exposed East China Sea during the LGM connected populations in eastern China, the Korean Peninsula and southern Japan. Relatively high distribution probabilities were predicted in some disjunctive areas including southernmost of Korean Peninsula with part of East China Sea (ECS) basin, north of Yangtze river area, south of Daba Mountains, and continuous areas including southern Japan and southern China.

## Discussion

### Distribution Characteristics of Chloroplast Haplotypes

Haplotype variation has been studied for several plant species in East Asia and China including *Pinus tabulaeformis* [12], *Ostryopsis davidiana* [13], *Kirengeshoma* [14], *Dysosma versipellis* [15] and *Taxus wallichiana* [55]. In this study, we found that *Q. variabilis* had 26 haplotypes detected, with possible 114 missing ones and three un-sampled or extinct ancestral ones, with a complicated phylogenetic network and a lacking of distinct geographic structure (Fig. 1, 2). Populations in Central-Eastern China had more haplotypes, while only one or two haplotypes appeared in the populations in peripheral areas (e.g., the northern and southern Mainland China, Korean Peninsula, and Zhoushan and Japanese Archipelagoes and the Taiwan Islands). Some haplotypes only appeared in population of several regions in general. Although no obvious phylogeographical structure existed (*P* = 0.422 for difference between *G*_ST_ and *N*_ST_, Table 3) from analyses for all populations combined, there is a closer relationship among haplotypes distributed in Central-Eastern China, based on the difference between *G*_ST_ and *N*_ST_ (P = 0.012). One important distribution characteristic is that many chloroplast haplotypes are discontinuous, such as H4 occurred in two locations which are more than 2,655 km apart, H5 is wide spread in eastern Asia but missing in most of populations in Central-Eastern China, H11 was found in coastal areas and islands but not in the inland regions except for Louguan Tai in Northwestern China. Another two cases of disjunct occurrence were found in H2 and H8. These disjunctions may be due to long-term fragmentation of *Q. variabilis* forests in the past. There was massive deforestation in East Asia, particularly in Central-Eastern and Northern China started around 1360s (start of the Ming Dynasty). There was also large scale clearing of forest areas for large agricultural expansion due to population expansion. This deforestation event resulted in large fragmentation of native forests and produced many isolated small plant populations [56]. From about the 1950s, there was also another round massive deforestation in Central-Eastern, Northern, and Southern and Northwestern regions which further defragmented large areas of Central-Eastern China. These anthropogenic disturbances may have directly caused the significant loss of haplotypes in several plant species, including *Q. variabilis*, as similar distribution characteristic also could be found in other species in the Chinese mainland [13], [55]–[57]. Other factors including seed or plant migration accompanied through human migration and civil wars could not be ruled out.

Different haplotypes were detected in the adjacent populations from the same region. For example, H1 and H8 occurred in the North and South of Yangtze River respectively, where Yangtze River acted as a geographic barrier. In Laotung Peninsula, H6 and H8 were found in two close populations which might be derived from separate refugia.

### Genetic Diversity and Population Differentiation

We detected a high level of total genetic diversity (*H*_T_ = 0.888) across the 50 populations of *Q. variabilis* in East Asia. The long evolutionary history of the species may have accumulated such genetic diversity. The wide geographical range of this species across a geologically dynamic region may help to provide ample opportunity for isolation, drift and conservation of the mutated alleles [58]. Molecular variance analysis showed that genetic diversity of *Q. variabilis* was higher among populations within regions (*H*_T_ = 0.667–1.00) and across the entire distribution, but lower within population (0–0.249) in these sampled regions (Table 3). This pattern of chloroplast genetic variation was similar to that observed in other species distributed in the same range and nearby regions. For example, *Ostryopsis davidiana* in Northwestern and North China [13] and *Taxus wallichiana* in southern China and northern Vietnam [55] showed similar patterns. Population genetic differentiation index (*G*_ST_) indicated large population differences. There was higher genetic differentiation between the populations in Mainland China and populations in Zhoushan and Japanese Archipelagoes, Taiwan Islands, and Korea Peninsula. This observed large differentiation could be readily attributed to isolation by seas. The abundance diversity among populations could also due to increased genetic drift in smaller populations, broad ecological niches, besides isolation by distance or barrier by local mountains. As an example, Oriental oak produce big-sized oak acorns which are dispersed mainly by rodents such as *Apodemus peninsulae*, *Niviventor confucianus* and *A. agrarius* over limited distances [59]. *Q. variabilis* is also a monoecious, autophilous plants, pollinated by wind and insects. Previous molecular studies have suggested that no species-specific nucleotide differences in several chloroplast DNA intergenic spacers in Japanese oak species owing to hybridization and (or) shared ancient polymorphism [60], [61]. In this study, we detected no intra-species genetic diversity in six *Q. variabilis* Japanese populations. One possible explanation is that these populations might have gone through a bottleneck effect or founder effect when the population from mainland migrated to the Japan Archipelago. Similar phenomenon could also occur in Taiwan Island and Korea Peninsula. The similarity among Japan Archipelago, Taiwan Island and Korea Peninsula populations could be supported by the affinitive and shared haplotypes between Islands, simulated palaeodistribution and population expansion analysis.

### Phylogenetic Relationships and Potential Refugia

Glacial refugia were assumed to harbor high diversity of genotypes and major lineages within a species [2], [62]. Coalescent theory predicts that older alleles will occupy interior nodes of a haplotype network [63] and these haplotypes would have a great probability of producing mutational derivatives and may represent some relict ancestral genotypes [64]. Widespread and predominant haplotypes also were suggested to be older haplotypes [65]. Because of unclear relationships among the many haplotypes observed in this study, it is difficult to interpret their evolution history and their ancestral position. However, the network shows a central position for the widespread haplotype H5, which may represent an ancestral haplotype. By considering the Bayesian tree and network map, one may postulate that H2 and H11 could be more ancient old haplotypes. Based on observed cpDNA haplotypes and nucleotide diversity of *Q. variabilis* populations (Information S1), it may be possible that multiple glacial refugia existed in the Central-Eastern China with the current Oriental oak distribution range. XY population in Dabie Mountains and HY population in the west of Hubei had the highest haplotype diversity and nucleotide diversity and could be candidate places for glacial refugia at the LGM time. Our hypotheses of multiple refugia also support a previous study using cpSSR and PCR-RFLP markers [66]. The strong predictions for occurrence during LGM period coupled with high haplotype diversity suggest that long-term climate stability has maintained relatively high genetic diversity in this region.

Population AK in Northwestern China, located between Qinling and Daba Mountains, a region well-known as a large natural biological gene pool for many species had high level nucleotide diversity and could be another candidate as a refugial location. YB population also had relative high genetic diversity, and located within the ancient continuous distribution of *Q. variabilis* population in south China according to ENM data analysis. This, coupled with fossil record of deciduous *Quercus* species in the adjacent areas during Pliocene [67], may also indicate the presence of *Q. variabilis* refugia.

Whether population PG which had the second highest nucleotide diversity (*π = *1.02×10^−3^) could represent a remnant of an ancient centre of diversity for the species in North China region is an interesting observation. Previous investigations on conifer species preferred refugia in northern China during Pleistocene glaciations [12], [17], [68]. According to Harrison et al. [10], climatic condition during the LGM has confined the temperate forests to the medium elevations in northern China, while taiga and non-forest occupied northern and northeast China (above 30°N). In this study, we constructed the paleodistribution of *Q. variabilis* with the range extended above 30°N and below 40°N, a finding that is somewhat inconsistent to the inference by Harrison et al., but consistent with Qian and Ricklefs’ hypothesis [11]. This meant our findings support the assumption that the area inhabited by population PG could be a center of colonization or secondary diversification for *Q. variabilis*.

Population CN in the south of Korea Peninsula was found to have relative high genetic diversity and ENM analysis also showed a possibility of a glacial refuge there. Molecular data of several other species including other fagaceous plants had proposed a primary diversity centre in central Taiwan and the west of the Taiwan’s Central Mountain Ridge [69]. We detected a low level of genetic variation in Taiwan Island, but the population TN (Nantou, Taiwan) had some genetic diversity, indicating a possibility as Taiwan’s refuge. Southern Japan could be a possible refuge region during LGM period, as we detected absent genetic diversity in Japan’s populations of *Q. variabilis*, with palaeodistribution modelling predicted suitable climate there for the species. A possible bottleneck effect of population reduction and the subsequent separation from mainland and other Islands could be a responsible explanation for such low diversity.

### Historical Demography in Response to Climatic Oscillations

Typical responses of plants to climate changes were adaptive evolution through migration, resulting in the alteration of geographical distribution range [3], [4]. The current distribution range of *Q. variabilis* covers MAT from 7°C in the northern China to 23°C in the southern China, and about 15°C in the central China [70]. It’s likely that a southward shift of *Q. variabilis* distribution from more than 40°N (Liaoning province) to less than 35°N (Anhui Province) comparing population distribution at present and during LGM, inferred from our ENM construction. With pollen record in the sediment profiles collected in Taihu Lake (31° 30′ N, 120° 30′ E), Xu et al. showed that in Taihu Lake, before 11000–9000 BP, deciduous broadleaf species (especially Fagaceae, including *Quercus* spp.) predominated while conifer species (e.g., *Pinus* spp.) co-existed [71]. Both unimodal mismatch distribution and significant negative Fu’s *F_S_* indicated a demographic expansion of Oriental oak populations in East Asia. The results were consistent with Bayesian skyline plot that the ascent curve indicated an increased population size in Middle Pleistocene, a period that saw the advance and retreat of glaciers numerous times. Based on our fossil-calibrated cpDNA phylogenies, a rapid speciation event occurred at late Quaternary and we traced the spatial expansion of *Q. variabilis* populations in East Asia to the last glacial cycle(s).

Before the LGM, *Q. variabilis* may have distributed across the temperate area in East Asia, with the northern range to Liaoning province at 40°N at least and the south range to the northern subtropical area at about 30°N. With the climate cooling at the beginning of the Quaternary, the north range of Oriental oak distribution retracted southwards (reaching to about 30°N) while some populations (e.g., Korean Peninsula) might remained in certain special geographical locations between 30 and 40°N as possible refugia. The populations in the southern tip of the distribution colonized southwards (reaching about 20°N), forming a deciduous broadleaf forests in the areas where current evergreen broadleaf forests grew. As the starting point of diversification is given at the Pleistocene with 1.45 Ma, this distribution changing was very likely to cause the divergence between ‘Island’ clades and ‘Inland’ clades.

At the peak of the LGM, the MAT was about 15°C in the south range of the distribution, and about 7°C in the central part (southern Henan and northern Hubei) of the current distribution area of *Q. variabilis* [8], deciduous forests prevailed in the central and south parts of Mainland China, with *Q. variabilis* as a common tree species at the time. Across the East China Sea, the continental shelf areas as the land-bridge connected the Mainland China and Taiwan Islands, Oriental oak could distributed across the continental shelf areas and Islands.

Although there have been a lot of literature showing a close relationship in flora distribution between Taiwan and Mainland China [29], [30], only few studies had focused on genetic relationship between the populations for a specific species [15]. Our results indicate certain genetic relationship in the oak populations in eastern China Mainland, Taiwan and Japan (Fig. 1). The affinity among haplotypes found in Mainland China, Korea Peninsular and Japan Archipelago could be traced back to Quaternary, during Pleistocene periods of lowered sea level associated with glacial maxima. At that time, the Yellow Sea was largely drained and these areas were connected by dry land with a climate suitable for temperate forest [11], [52], [54].

With increasing temperature after the LGM, *Q. variabilis* populations migrated northwards from Central-Eastern China, re-colonized these lost distribution range, and finally, reached the Liaoning area as the north edge of the current distribution. There is possibility that some populations in Liaotung Peninsular could be the descendant from southern Korea Peninsula refugia. While the sea level rose, the land-bridge disappeared and the sea water separated populations remaining in Japanese and Zhoushan Archipelagoes and Taiwan Island from Mainland China. The lower level of population differentiation could be explained by a strong bottleneck or founder effects during the separation of Mainland and Islands. Meanwhile, in southern part of subtropical areas, other evergreen trees became dominant species in the new forests and Oriental oak retreated and only become a companion tree in the current evergreen forests.

This might indicate that *Q. variabilis* had a different evolutionary history relative to *Q. robur* in Europe that *Q. robur* only survived in several refugia in Mediterranean areas at the LGM [72], while *Q. variabilis* would more likely have been preserved in more places in East Asia at the LGM. We conclude that Oriental oak might have colonized current distribution by northward recolonization and possible expansion of many refugia after the LGM. Deciduous Oriental oak, one of the most widely distributed plant in Asia, providing an ideal species for investigating the biogeography of plants across the Mainland China, Korea Peninsular, Zhoushan and Japanese Archipelagoes and the Taiwan Islands in relation to Quaternary climate using phylogeographical approaches. Oriental oak haplotypes constituted one of the most complicated phylogenetic network among the plants studied so far in the eastern Asia, with possible two distinct clades. The first clade of ‘Island’ is mainly consisted by populations in isolated Taiwan Islands, Zhoushan and Japanese Archipelagoes, and Central-Eastern coastal region of Mainland China. Populations in the second clade were mainly from Central and Western Mainland China. Our results showed that the Central-Eastern China had populations with more haplotypes, the highest nucleotide diversity and significant phylogeographical structure and was the main glacial refugia at last glacier ages. *Q. variabilis* was distributed as dominant tree in the areas of current evergreen forests in the Mainland China, and the continental shelf areas as the land-bridge between the Mainland and Islands of East China Sea at the LGM while its offshore refugia probably existed in southernmost Korea. Restricted seed dispersal mechanisms, geographical obstacles and human-induced fragmentation resulted in higher interpopulation differentiation across the current distribution range, and in particular, between the populations in the Mainland China and Archipelagoes and Taiwan Islands. Our findings supported the hypothesis that *Q. variabilis* had survived in scattered multiple glacial refugia in East Asia and experienced a range expansion and rapid speciation in response to climatic fluctuations during Quaternary.

## Supporting Information

Information S1 **3** **tables.**
(DOCX)
