# Supplementary material for: Phylogeography of Quercus variabilis Based on Chloroplast DNA Sequence in East Asia: Multiple Glacial Refugia and Mainland-Migrated Island Populations
Source: PLoS One. 2012 Oct 24;7(10):e47268. doi: 10.1371/journal.pone.0047268 (PMC3480369; doi:10.1371/journal.pone.0047268)
Supplement: Information S1 — 3 tables. (DOCX) [file pone.0047268.s001.docx]

**Supporting Information S1:**

**Table S1.** Universal Primers used for primer screening in the experiment.

|  | Primers | Sequence (5' to 3') | *T*_m_ | Variation Sites | Reference |
| --- | --- | --- | --- | --- | --- |
| 1 | trnT-trnL | F: CATTACAAATGCGATGCTCT  R: TCTACCGATTTCGCCATATC | 53.7  55.75 | 1 | Taberlet et al. 1991 [45] |
| 2 | trnL | F: CGAAATCGGTAGACGCTACG  R: GGGGATAGAGGGACTTGAAC | 59.85  59.85 | 0 | Taberlet et al. 1991 [45] |
| 3 | **trnL-trnF** | F: GGTTCAAGTCCCTCTATCCC  R: ATTTGAACTGGTGACACGAG | 59.85  55.75 | **4** | Taberlet et al. 1991 [45] |
| 4 | trnH(GUG)-psbA | F: ACTGCCTTGATCCACTTGGC  R: CGAAGCTCCATCTACAAATGG | 59.85  58.01 | F^※^ | Hamilton 1999 [46] |
| 5 | trnS(GCU)-trnG(UCC) | F: GCCGCTTTAGTCCACTCAGC  R: GAACGAATCACACTTTTACCAC | 61.9  56.35 | 11^▲^ | Hamilton 1999 [46] |
| 6 | psbB-psbF | F: GTTTACTTTTGGGCATGCTTCG  R: CGCAGTTCGTCTTGGACCAG | 58.21  61.9 | 0 | Hamilton 1999 [46] |
| 7 | rpl20-5'-rps12 | F: TTTGTTCTACGTCTCCGAGC  R: GTCGAGGAACATGTACTAGG | 57.8  57.8 | 0 | Hamilton 1999 [46] |
| 8 | **atpB-rbcL** | F: TAGTTTCTGTTTGTGGTGACAT  R: AAGTAGTAGGATTGGTTCTCAT | 52.54  52.54 | **8** | Terachi 1993 [47] |
| 9 | **trnH-psbA** | F: ACGGGAATTGAACCCGCGCA  R:TATTATTAACCGTGCTAACC | 59.5  49.25 | **9** | Okaura et al. 2007 [48] |
| 10 | trnV-trnM | F: GCTATACGGGCTCGAACC  R: TACCTACTATTGGATTTGAACC | 58  52.54 | 1 | Hwang et al. 2002 [49] |
| 11 | petG-trnP | F: GGTCTAATTCCTATAACTTTGGC  R: GGGATGTGGCGCAGCTTGG | 54.86  64 | 0 | Hwang et al. 2002 [49] |
| 12 | trnQ-trnS | F: CGGAAGGATTCGAACCTC  R: AGTAAGCATTACACAATCTCCAA | 56  53.07 | 1 | Kanno et al. 2004 [50] |

Note: ※ indicates the sequencing was failed; ▲ indicates it won't show in the results because of short samples succeed in sequencing to align with other three fragments

**Table S2.** Various nucleotide sites of chloroplast *trn*L-*trn*F, *atp*B-*rbc*L, *trn*H-*psb*A sequences of the 50 *Q. variabilis* populations sampled across eastern Asia.

|  | *n* | *trn*L-*trn*F | | | | *atp*B-*rbc*L | | | | | | | | | *trn*H-*psb*A | | | | | | | | | |
| --- | --- | --- | --- | --- | --- | --- | --- | --- | --- | --- | --- | --- | --- | --- | --- | --- | --- | --- | --- | --- | --- | --- | --- | --- |
|  |  | 91 | 103 | 202-206 | 252 | 210 | 216 | 220 | 254 | 293 | 349 | 350 | 367 | 443 | 110-130 | 253 | 266 | 267 | 354-374 | 375-395 | 401 | 403-410 | 411-434 | 479 |
| H1 | 23 | A | - | ▲_1_ | T | T | A | G | T | G | A | - | C | A | - | T | A | - | - | - | C | ▲_4_ | - | G |
| H2 | 47 | . | - | ▲_1_ | . | . | . | . | . | . | . | - | . | . | - | . | . | - | - | - | A | ▲_4_ | - | . |
| H3 | 45 | . | T | ▲_1_ | G | . | . | . | . | . | . | - | . | . | - | . | . | - | - | - | A | - | - | A |
| H4 | 5 | . | T | ▲_1_ | G | . | . | . | . | . | . | - | T | . | - | . | . | - | - | - | A | ▲_4_ | - | . |
| H5 | 68 | . | - | ▲_1_ | G | . | . | . | . | . | . | - | . | . | - | . | . | - | - | - | A | ▲_4_ | - | . |
| H6 | 23 | . | T | ▲_1_ | G | . | . | . | . | . | . | - | . | G | - | . | . | - | - | - | A | ▲_4_ | - | . |
| H7 | 15 | . | T | ▲_1_ | G | . | . | . | . | . | . | - | T | . | - | . | . | - | ▲_3_ | - | A | ▲_4_ | - | . |
| H8 | 28 | . | - | ▲_1_ | G | . | . | . | C | . | . | - | T | . | - | . | - | - | - | - | A | ▲_4_ | - | . |
| H9 | 33 | . | - | - | G | . | . | T | . | . | . | - | T | . | - | . | . | - | - | - | A | ▲_4_ | - | . |
| H10 | 3 | . | T | ▲_1_ | G | . | . | . | . | . | . | A | . | . | - | . | . | - | - | - | A | - | - | . |
| H11 | 134 | . | - | ▲_1_ | G | . | . | . | C | . | . | - | T | . | - | . | . | - | - | - | A | ▲_4_ | - | . |
| H12 | 19 | . | - | ▲_1_ | G | . | . | . | . | . | - | - | . | . | - | . | . | - | - | - | A | ▲_4_ | - | . |
| H13 | 1 | . | - | ▲_1_ | G | . | . | . | . | . | - | - | . | . | - | . | - | - | - | - | A | ▲_4_ | - | . |
| H14 | 14 | . | T | ▲_1_ | G | . | . | . | . | . | . | - | T | . | ▲_2_ | . | . | - | - | - | A | ▲_4_ | - | . |
| H15 | 25 | - | T | ▲_1_ | G | . | . | . | . | . | . | - | . | . | - | . | . | - | - | - | A | ▲_4_ | - | . |
| H16 | 1 | . | - | ▲_1_ | . | . | . | . | C | . | . | - | T | . | - | . | . | - | - | - | A | ▲_4_ | - | . |
| H17 | 1 | . | T | ▲_1_ | G | . | . | . | . | . | . | - | . | . | - | . | . | - | - | - | A | ▲_4_ | - | . |
| H18 | 1 | . | - | ▲_1_ | G | . | . | . | . | . | . | - | T | . | - | . | . | - | ▲_3_ | - | A | ▲_4_ | - | . |
| H19 | 1 | . | - | ▲_1_ | . | . | . | T | . | . | . | - | T | . | - | . | . | - | - | - | A | ▲_4_ | - | . |
| H20 | 1 | . | - | ▲_1_ | G | . | . | . | C | . | . | - | T | . | - | . | . | A | - | - | A | ▲_4_ | - | . |
| H21 | 2 | . | - | ▲_1_ | G | . | . | . | . | . | . | - | T | . | - | . | . | - | - | - | A | ▲_4_ | - | . |
| H22 | 10 | . | - | ▲_1_ | G | . | C | . | . | . | . | - | . | . | - | . | . | - | - | - | A | ▲_4_ | ▲_5_ | . |
| H23 | 12 | . | - | ▲_1_ | G | . | . | . | . | . | . | - | T | . | - | . | . | - | ▲_3_ | ▲_3_ | A | ▲_4_ | - | . |
| H24 | 12 | . | - | ▲_1_ | G | . | . | . | C | . | . | - | T | . | - | G | . | - | - | - | A | ▲_4_ | - | . |
| H25 | 1 | . | - | ▲_1_ | G | C | . | . | . | . | . | - | . | . | - | . | . | - | - | - | A | ▲_4_ | - | . |
| H26 | 3 | . | T | ▲_1_ | G | . | . | . | . | C | . | - | T | . | - | . | . | - | - | - | A | ▲_4_ | - | . |

Note: sequences are numbered from the 5’ end to the 3’ end in each region. Dot (.) indicates that the character states are the same as Haplotype 1, ‘–’ indicates alignment gap. ▲_1_: ATTTC; ▲_2_: GACAAGAGGCAGAAAATAAAT; ▲_3_: TATAATAATTTAATAATTAAA; ▲_4_: TAAATTAA; ▲_5_: TAAATAAGAAAGCTAATAAATTAA.

**Table S3.** The distribution of the 26 haplotypes in the 50 *Q. variabilis* populations based on three non-coding regions in cpDNA.

| **Code** | **H1** | | **H2** | | **H3** | | **H4** | | **H5** | | **H6** | | **H7** | | **H8** | | **H9** | | **H10** | | **H11** | | **H12** | | **H13** | | **H14** | | **H15** | | **H16** | | **H17** | | **H18** | | **H19** | | **H20** | | **H21** | | **H22** | | **H23** | | **H24** | | **H25** | | **H26** | | **Total** | |  |
| --- | --- | --- | --- | --- | --- | --- | --- | --- | --- | --- | --- | --- | --- | --- | --- | --- | --- | --- | --- | --- | --- | --- | --- | --- | --- | --- | --- | --- | --- | --- | --- | --- | --- | --- | --- | --- | --- | --- | --- | --- | --- | --- | --- | --- | --- | --- | --- | --- | --- | --- | --- | --- | --- | --- | --- |
| **SY** |  | |  | |  | |  | |  | |  | |  | |  | |  | |  | | **10** | |  | |  | |  | |  | |  | |  | |  | |  | |  | |  | |  | |  | |  | |  | |  | | **10** | |  |
| **LD** |  | |  | |  | |  | |  | |  | |  | | **10** | |  | |  | |  | |  | |  | |  | |  | |  | |  | |  | |  | |  | |  | |  | |  | |  | |  | |  | | **10** | |  |
| **LZ** |  | |  | |  | |  | |  | | **17** | |  | |  | |  | |  | |  | |  | |  | |  | |  | |  | |  | |  | |  | |  | |  | |  | |  | |  | |  | |  | | **17** | |  |
| **CN** |  | |  | |  | |  | |  | | **6** | | **3** | |  | |  | |  | |  | |  | |  | |  | |  | |  | |  | |  | |  | |  | |  | |  | |  | |  | |  | |  | | **9** | |  |
| **KC** |  | |  | |  | |  | |  | |  | | **12** | |  | |  | |  | |  | |  | |  | |  | |  | |  | |  | |  | |  | |  | |  | |  | |  | |  | |  | |  | | **12** | |  |
| **KK** |  | |  | |  | |  | | **8** | |  | |  | |  | |  | |  | |  | |  | |  | |  | |  | |  | |  | |  | |  | | **1** | |  | |  | |  | |  | |  | |  | | **9** | |  |
|  |  | |  | |  | |  | |  | |  | |  | |  | |  | |  | |  | |  | |  | |  | |  | |  | |  | |  | |  | |  | |  | |  | |  | |  | |  | |  | |  | |  |
| **HX** |  | |  | |  | |  | | **1** | |  | |  | |  | |  | |  | |  | |  | |  | |  | | **11** | |  | |  | |  | |  | |  | |  | |  | |  | |  | |  | |  | | **12** | |  |
| **TL** |  | |  | | **10** | |  | |  | |  | |  | |  | |  | |  | |  | |  | |  | |  | |  | |  | |  | |  | |  | |  | |  | |  | |  | |  | |  | |  | | **10** | |  |
| **HYS** |  | |  | |  | |  | |  | |  | |  | |  | |  | |  | |  | |  | |  | |  | | **12** | |  | |  | |  | |  | |  | |  | |  | |  | |  | |  | |  | | **12** | |  |
| **PG** |  | |  | | **4** | |  | |  | |  | |  | |  | |  | |  | |  | |  | |  | |  | |  | |  | |  | |  | |  | |  | |  | |  | |  | | **12** | |  | |  | | **16** | |  |
|  |  | |  | |  | |  | |  | |  | |  | |  | |  | |  | |  | |  | |  | |  | |  | |  | |  | |  | |  | |  | |  | |  | |  | |  | |  | |  | |  | |  |
| **AK** |  | | **3** | | **9** | |  | |  | |  | |  | |  | |  | |  | |  | |  | |  | |  | |  | |  | |  | |  | |  | |  | |  | |  | |  | |  | |  | |  | | **12** | |  |
| **TGB** |  | |  | |  | |  | |  | |  | |  | |  | |  | |  | |  | | **4** | |  | |  | |  | |  | |  | |  | |  | |  | |  | |  | |  | |  | |  | |  | | **4** | |  |
| **NY** |  | |  | |  | |  | | **1** | |  | |  | |  | |  | |  | |  | |  | |  | |  | |  | |  | |  | |  | |  | |  | |  | |  | | **12** | |  | |  | |  | | **13** | |  |
| **LGT** |  | |  | |  | |  | |  | |  | |  | |  | |  | |  | | **8** | |  | |  | |  | |  | |  | |  | |  | |  | |  | |  | |  | |  | |  | |  | |  | | **8** | |  |
| **TB** |  | | **10** | |  | |  | |  | |  | |  | |  | |  | |  | |  | |  | |  | |  | |  | |  | |  | |  | |  | |  | |  | |  | |  | |  | |  | |  | | **10** | |  |
| **SMX** |  | |  | | **6** | |  | |  | |  | |  | |  | |  | |  | |  | |  | |  | |  | |  | |  | |  | |  | |  | |  | |  | |  | |  | |  | |  | |  | | **6** | |  |
| **BMT** |  | |  | |  | |  | | **7** | |  | |  | |  | |  | |  | |  | |  | |  | |  | |  | |  | |  | |  | |  | |  | |  | |  | |  | |  | |  | |  | | **7** | |  |
| **GT** |  | | **12** | |  | |  | | **1** | |  | |  | |  | |  | |  | |  | |  | |  | |  | |  | |  | |  | |  | |  | |  | |  | |  | |  | |  | |  | |  | | **13** | |  |
|  |  | |  | |  | |  | |  | |  | |  | |  | |  | |  | |  | |  | |  | |  | |  | |  | |  | |  | |  | |  | |  | |  | |  | |  | |  | |  | |  | |  |
| **JX** |  | | **9** | |  | |  | |  | |  | |  | |  | |  | |  | | **3** | |  | |  | |  | |  | |  | |  | |  | | **1** | |  | |  | |  | |  | |  | |  | |  | | **13** | |  |
| **CW** |  | |  | |  | |  | |  | |  | |  | | **8** | |  | |  | |  | |  | |  | |  | |  | |  | |  | |  | |  | |  | |  | |  | |  | |  | |  | |  | | **8** | |  |
| **HZ** |  | |  | |  | | **1** | | **7** | |  | |  | |  | |  | | **1** | |  | |  | |  | |  | |  | |  | |  | | **1** | |  | |  | |  | |  | |  | |  | |  | |  | | **10** | |  |
| **HY** |  | | **5** | | **1** | |  | |  | |  | |  | |  | |  | |  | |  | | **3** | |  | |  | |  | | **1** | | **1** | |  | |  | |  | |  | |  | |  | |  | |  | |  | | **11** | |  |
| **BMH** |  | |  | | **13** | | **1** | |  | |  | |  | |  | |  | |  | |  | |  | |  | |  | |  | |  | |  | |  | |  | |  | |  | |  | |  | |  | |  | |  | | **14** | |  |
| **MS** |  | |  | |  | |  | |  | |  | |  | |  | |  | |  | |  | |  | |  | | **1** | |  | |  | |  | |  | |  | |  | | **1** | | **10** | |  | |  | |  | |  | | **12** | |  |
| **FJY** |  | |  | |  | |  | |  | |  | |  | |  | |  | |  | |  | | **12** | | **1** | |  | |  | |  | |  | |  | |  | |  | |  | |  | |  | |  | |  | |  | | **13** | |  |
| **XY** |  | |  | | **2** | |  | |  | |  | |  | |  | |  | |  | |  | |  | |  | |  | | **2** | |  | |  | |  | |  | |  | |  | |  | |  | |  | | **1** | | **3** | | **8** | |  |
| **NJ** |  | |  | |  | |  | |  | |  | |  | | **10** | |  | |  | |  | |  | |  | |  | |  | |  | |  | |  | |  | |  | |  | |  | |  | |  | |  | |  | | **10** | |  |
| **AF** | **12** | |  | |  | |  | |  | |  | |  | |  | |  | |  | |  | |  | |  | |  | |  | |  | |  | |  | |  | |  | |  | |  | |  | |  | |  | |  | | **12** | |  |
| **AX** | **11** | |  | |  | |  | |  | |  | |  | |  | |  | |  | |  | |  | |  | |  | |  | |  | |  | |  | |  | |  | |  | |  | |  | |  | |  | |  | | **11** | |  |
| **DX** | |  | |  | |  | |  | | **7** | |  | |  | |  | |  | |  | |  | |  | |  | |  | |  | |  | |  | |  | |  | |  | |  | |  | |  | |  | |  | |  | | **7** | |
| **FD** | |  | |  | |  | |  | |  | |  | |  | |  | |  | |  | | **12** | |  | |  | |  | |  | |  | |  | |  | |  | |  | |  | |  | |  | |  | |  | |  | | **12** | |
| **GD** | |  | |  | |  | |  | |  | |  | |  | |  | |  | |  | | **3** | |  | |  | |  | |  | |  | |  | |  | |  | |  | |  | |  | |  | |  | |  | |  | | **3** | |
|  | |  | |  | |  | |  | |  | |  | |  | |  | |  | |  | |  | |  | |  | |  | |  | |  | |  | |  | |  | |  | |  | |  | |  | |  | |  | |  | |  | |
| **GX** | |  | |  | |  | |  | | **11** | |  | |  | |  | |  | |  | |  | |  | |  | |  | |  | |  | |  | |  | |  | |  | |  | |  | |  | |  | |  | |  | | **11** | |
| **YA** | |  | |  | |  | |  | | **15** | |  | |  | |  | |  | |  | |  | |  | |  | |  | |  | |  | |  | |  | |  | |  | |  | |  | |  | |  | |  | |  | | **15** | |
| **YB** | |  | |  | |  | | **3** | | **8** | |  | |  | |  | |  | |  | |  | |  | |  | |  | |  | |  | |  | |  | |  | |  | | **1** | |  | |  | |  | |  | |  | | **12** | |
| **YL** | |  | | **8** | |  | |  | |  | |  | |  | |  | |  | |  | |  | |  | |  | |  | |  | |  | |  | |  | |  | |  | |  | |  | |  | |  | |  | |  | | **8** | |
| **HH** | |  | |  | |  | |  | | **1** | |  | |  | |  | |  | |  | |  | |  | |  | | **13** | |  | |  | |  | |  | |  | |  | |  | |  | |  | |  | |  | |  | | **14** | |
|  | |  | |  | |  | |  | |  | |  | |  | |  | |  | |  | |  | |  | |  | |  | |  | |  | |  | |  | |  | |  | |  | |  | |  | |  | |  | |  | |  | |
| **DM** | |  | |  | |  | |  | |  | |  | |  | |  | | **9** | | **2** | |  | |  | |  | |  | |  | |  | |  | |  | |  | |  | |  | |  | |  | |  | |  | |  | | **11** | |
| **ZP** | |  | |  | |  | |  | |  | |  | |  | |  | | **12** | |  | |  | |  | |  | |  | |  | |  | |  | |  | |  | |  | |  | |  | |  | |  | |  | |  | | **12** | |
| **ZY** | |  | |  | |  | |  | |  | |  | |  | |  | | **12** | |  | |  | |  | |  | |  | |  | |  | |  | |  | |  | |  | |  | |  | |  | |  | |  | |  | | **12** | |
| **TN** | |  | |  | |  | |  | | **1** | |  | |  | |  | |  | |  | | **5** | |  | |  | |  | |  | |  | |  | |  | |  | |  | |  | |  | |  | |  | |  | |  | | **6** | |
| **TK** | |  | |  | |  | |  | |  | |  | |  | |  | |  | |  | | **9** | |  | |  | |  | |  | |  | |  | |  | |  | |  | |  | |  | |  | |  | |  | |  | | **9** | |
| **TT** | |  | |  | |  | |  | |  | |  | |  | |  | |  | |  | | **11** | |  | |  | |  | |  | |  | |  | |  | |  | |  | |  | |  | |  | |  | |  | |  | | **11** | |
| **TH** | |  | |  | |  | |  | |  | |  | |  | |  | |  | |  | | **14** | |  | |  | |  | |  | |  | |  | |  | |  | |  | |  | |  | |  | |  | |  | |  | | **14** | |
| **JY** | |  | |  | |  | |  | |  | |  | |  | |  | |  | |  | | **7** | |  | |  | |  | |  | |  | |  | |  | |  | |  | |  | |  | |  | |  | |  | |  | | **7** | |
| **JT** | |  | |  | |  | |  | |  | |  | |  | |  | |  | |  | | **11** | |  | |  | |  | |  | |  | |  | |  | |  | |  | |  | |  | |  | |  | |  | |  | | **11** | |
| **JH** | |  | |  | |  | |  | |  | |  | |  | |  | |  | |  | | **12** | |  | |  | |  | |  | |  | |  | |  | |  | |  | |  | |  | |  | |  | |  | |  | | **12** | |
| **JK** | |  | |  | |  | |  | |  | |  | |  | |  | |  | |  | | **12** | |  | |  | |  | |  | |  | |  | |  | |  | |  | |  | |  | |  | |  | |  | |  | | **12** | |
| **JG** | |  | |  | |  | |  | |  | |  | |  | |  | |  | |  | | **8** | |  | |  | |  | |  | |  | |  | |  | |  | |  | |  | |  | |  | |  | |  | |  | | **8** | |
| **JN** | |  | |  | |  | |  | |  | |  | |  | |  | |  | |  | | **9** | |  | |  | |  | |  | |  | |  | |  | |  | |  | |  | |  | |  | |  | |  | |  | | **9** | |
| **Total** | | **23** | | **47** | | **45** | | **5** | | **68** | | **23** | | **15** | | **28** | | **33** | | **3** | | **134** | | **19** | | **1** | | **14** | | **25** | | **1** | | **1** | | **1** | | **1** | | **1** | | **2** | | **10** | | **12** | | **12** | | **1** | | **3** | | **528** | |
| **Frequency** | | **0.044** | | **0.089** | | **0.085** | | **0.009** | | **0.129** | | **0.044** | | **0.028** | | **0.053** | | **0.063** | | **0.006** | | **0.254** | | **0.036** | | **0.002** | | **0.027** | | **0.047** | | **0.002** | | **0.002** | | **0.002** | | **0.002** | | **0.002** | | **0.004** | | **0.019** | | **0.023** | | **0.023** | | **0.002** | | **0.006** | | **1** | |
| **Population** | | **2** | | **6** | | **7** | | **3** | | **12** | | **2** | | **2** | | **3** | | **3** | | **2** | | **15** | | **3** | | **1** | | **2** | | **3** | | **1** | | **1** | | **1** | | **1** | | **1** | | **2** | | **1** | | **1** | | **1** | | **1** | | **1** | |  | |
